# The risk of prematurity in Cameroonian children born after in vitro fertilisation

**DOI:** 10.4314/gmj.v57i2.6

**Published:** 2023-06

**Authors:** Aimée-Sandrine F. Nzenti, Christopher Aimakhu, Paul N. Koki, Teshome Gensa, Massa Sakouvogui

**Affiliations:** 1 Department of Pediatrics, University of Yaoundé 1, Cameroon; 2 Pan African University Institute for Life and Earth Sciences (Including Health and Agriculture), PAULESI, University of Ibadan, Ibadan, Nigeria; 3 Department of Obstetrics and Gynecology, College of Medicine, University College Hospital, University of Ibadan, Nigeria

**Keywords:** In vitro-fertilization, prematurity, risk, Cameroon

## Abstract

**Objective:**

To evaluate the risk of prematurity in Cameroonian children born after in vitro Fertilisation.

**Design:**

A retrospective cohort study.

**Setting:**

Conducted at the pediatric department of the Hospital Center for Research and Application in Endoscopic Surgery and Human Reproduction (HCRAESHR) in Yaoundé over eight months.

**Participants:**

Every newborn born after in vitro fertilisation (exposed group) and those born after spontaneous conception (non-exposed group) from a singleton pregnancy were included. Multiple pregnancies were excluded. One hundred newborns per group were recruited and matched according to the mode of delivery.

**Interventions:**

The main outcome measure was prematurity at birth. Data were collected from the medical records of the newborns and reported on individual questionnaires. The t Student test was used to assess the differences in gestational age between the two groups. The generalised linear model using binomial probability distribution was used for multivariate analysis to determine prematurity risk factors. All results with a p-value ≤ 0.05 were considered statistically significant.

**Results:**

Prematurity was significantly predominant in the exposed group (22% and 5%, respectively, p=0.002) compared to the non-exposed group. The risk of prematurity in the exposed group was 4.4 times higher than in the non-exposed group. After controlling for confounders (the maternal age, the sex of the baby, and maternal hypertension), this risk increased significantly from 4.4 to 7.67 (p=0.001).

**Conclusion:**

This study demonstrated the first evidence from our part of the world showing that in vitro fertilisation is an absolute risk of prematurity.

**Funding:**

None declared

## Introduction

Couple infertility is a major public health issue.^1^ It is mainly reported among women aged 35 years and above. The prevalence of infertility continues to grow in Africa, and in Cameroon, it ranges from 20 to 30%.^2^ With the high prevalence of infertility among couples, assisted reproductive techniques (ART) is increasingly a treatment option for infertile couples. In vitro fertilisation (IVF) is one of the three main forms of artificial procreation. The others include artificial insemination (AI) and intra-cytoplasmic sperm injection (ICSI).^3^ In vitro fertilisation occurs in several stages, from ovarian stimulation, oocyte puncture, getting a zygote after fertilisation, cleavage and embryonic development and then embryonic transfer into the uterus.^4^

Several studies have been conducted on infertility and IVF globally. Most of these studies have focused on IVF's economic and psychosocial aspects and maternal complications after IVF. Very few of them have assessed the prevalence of premature births among women who underwent IVF. In Africa, studies based on the risk of neonatal prematurity after IVF are sparse. However, some authors have reported a prevalence of premature births after IVF at 13.5% in Nigeria^5^, 58.3% in Sudan^6^ and 10.9% in Mali.^7^ In Cameroon, there is a lack of studies considering premature births among women following IVF.

This retrospective cohort study was conducted to determine the prevalence of prematurity and evaluate its risk in newborns after in vitro-fertilization compared to those from spontaneous conception (without any medical assistance) in Yaoundé, Cameroon.

## Methods

### Study design

This study was a retrospective cohort study that lasted eight months, from February to September 2021. All newborns who met the inclusion criteria were recruited.

### Study setting

This study was conducted at the Hospital Center for Research and Application in Endoscopic Surgery and Human Reproduction (HCRAESHR) paediatrics department in Yaoundé, Cameroon. It is a public administrative establishment responsible which offers assisted reproductive technology services to women in the country. In addition, they provide high-level obstetrics and gynaecologic while conducting research into endoscopy and human reproduction. The hospital comprises a maternity unit, six labour and delivery rooms, a pediatric department (including a neonatology unit), and six operating rooms. In this hospital, all pregnancies from IVF, without exception, deliver by caesarean section. The hospital began offering IVF services in 2015. About 200 cases of IVF per year are reported. From March 2015 until October 2021, around 280 babies were born at the facility through the technique, with an average of 40 babies born from IVF per year. IVF is the only assisted reproductive technique used in this centre. During the IVF procedure, ovarian stimulation was performed either through natural or stimulation protocols. Stimulated protocols used products such as clomiphene citrate and exogenous gonadotropins (FSH and LH), allowing clinicians to remove the oocyte. In a natural cycle, the oocyte was removed before the LH peak occurs in the middle. In both protocols, when the oocyte matured, it was collected by the transvaginal aspiration for fertilisation. Afterwards, the resulting embryos were incorporated into the maternal uterus either in a fresh or frozen, thawed state.

### Participants

This study included all newborns from a singleton pregnancy after spontaneous conception and after IVF. All newborns from multiple pregnancies were excluded. The sample size was obtained using the formula from Serhier Z et al. 8 for cohort studies:


$$\text{N}=2\text{n}=\frac{2\text{[Zα}/2\times \sqrt{\left(2\text{P}\times (1-\text{P}\right)}+\text{Z}(1-\text{β})\times \;\sqrt{\text{P}1(1-\text{P}1)+\text{P}2(1-\text{P}2){]}^{2}}}{{\left(\text{P}1-\text{P}2\right)}^{2}}$$

Where N is the total sample size, n is the corresponding size in each group with equal size, Zα/2 is the normal deviation at a significant level (a), Z (1-β) is a normal deviation at a power (1-β) with a confidence interval β. Thus, α = 5%, with Zα/2= 2 and β= 95% with Z (1-β) = Z (0.05) = 1.6 were used in this study. P1 and P2 are the proportions of the anomalies observed respectively in the exposure and non-exposure groups obtained from previous studies, and P = (p1 + p2) / 2 is the average of these two proportions. However, due to the absence of studies on this topic in Cameroon, and after critically appraising the available literature, the current study used the prevalence obtained in the study of Traoré SO et al. in Mali. 7 The study highlighted neonatal prematurity in 2 groups, one from spontaneous pregnancies and one from IVF. Therefore, from the reference study: p1 = 28.4%, p2 = 12.3% and p = 20.3%. Then, N = 2×4/0.04 =200 participants, comprised of 100 newborns in the exposure group (born after IVF) and 100 newborns in the non-exposure group (from spontaneous conception) at HCRAESHR. During the data collection, we matched the two groups according to the mode of delivery, with a matching coefficient of 1.0.

### Procedure

Data were collected using the hard copy of the newborns' birth medical records, stored in that hospital's pediatric department. These data were reported on a structured questionnaire and covered IVF underwent, gestational age, delivery mode, gender, birth weight, maternal parity, abortion and diseases in pregnancy. Therefore, all children born before 37 weeks of gestational age, based on the clinical estimate, were considered as premature and all newborns with a birth weight under 2.5 kg were considered to have a low birth weight.^9^ No iatrogenic preterm delivery was carried out in this sample.

### Statistical analysis

The collected data were entered and analysed using SPSS (statistical package of social science) software version 23.0 and Excel 2013 for Windows version 13.0. Frequency tables were generated, and the two groups were compared using the Chi-square test, t Student test, and one-way ANOVA (analysis of variance). The relative risk was calculated to highlight the risk of prematurity in newborns after IVF without considering the effects of confounding factors like the sex of the baby, the maternal age and the maternal disease in pregnancy, such as hypertension. Statistical significance was set at p ≤0.05 with a 95% confidence interval and an error of 5%. The outcomes identified as significant at univariate analysis (with a p-value <0.2) were subjected to generalised linear models using binomial probability distribution while including potential confounding factors.

All variables without a significant result (p ≤0.05) were eliminated, and the model was repeated until only all the significant risk factors of prematurity remained.

### Ethical consideration

Ethical clearance was obtained from the University of Ibadan/University College Hospital, Ibadan (UI/UCH) Ethics Committee with the reference number UI/EC/20/0473.

## Results

### Socio-demographic characteristics of participants

All participants were Cameroonian. The two groups were homogeneous concerning their mode of delivery. Our sample comprised 40 males and 60 females in each group.

### Maternal characteristics

Mothers of newborns following IVF were significantly older at conception than those of newborns from spontaneous pregnancies (40.55 ± 8.35; CI: 38.89-42.21 and 35.72 ± 5.01 years; CI: 34.73-36.71, respectively) (p< 0.001). The minimum and maximum maternal ages were 26 and 61 years old in the exposed group compared to 23 and 49 years old in the non-exposed group. Moreover, the prevalence of primiparity and abortion was significantly predominant in the exposed group compared to the non-exposed group (20 % and 7%; p=0.007) for primiparity and 71% and 43%; p<0.001 for abortion).

In contrast, the prevalence of disease in pregnancy was homogenous in the two groups. These were arterial hypertension (12%), diabetes (7%), hepatitis B (4%), hepatitis C (3%), Sickle cell disease (5%) and acquired immune deficiency syndrome (AIDS) (1%).

### Mode of delivery

Newborns in the two groups were all born through cesarean section. In the exposed group, cesarean section was indicated for precious pregnancies due to IVF (infertility). In the non-exposed group, indications of cesarean sections were acute fetal distress (26%), double scar on the uterus (21%), contracted pelvis (17%), uterine rupture (3%), prolonged labour (8%), umbilical cord prolapse (4%), placenta praevia (3%), pre-eclampsia (4%), myomata of the uterus (4%) and breech presentation (10%).

### Premature births

Prematurity at birth was significantly predominant in newborns following IVF compared to those conceived spontaneously (22% and 5%, respectively; p= 0.002). Among these premature births, 11% from the exposed group weighted less than 2000g compared to 2% in the non-exposed group.

Furthermore, 10% of the exposed group weighted between 2000-2500g compared to 3% in the non-exposed group. Newborns in the exposed group had 4.4 times (CI: 1.73-11.16) higher risk of being born prematurely than those in the non-exposed group).

After controlling for the potential confounding factors such as their sex, maternal age and diseases in pregnancy like hypertension, the risk increased significantly, from 4.4 to 7.67 (p=0.001) (see Table 1).

**Table 1:** Adjusted relative risk (RR), predicting neonatal prematurity by selected variables (F= 200) (Constant:-4.96)

Variables	<37 weeks (F= 27)	≥ 37 weeks (F= 173)	Crude RR (95% CI)	Adjusted RR [95% CI]	B	P value
**1. IVF Yes**	22	78	**4.4** [1.74-11.16	**7.67** [2.41-24.42]	**2.04**	**0.001**
No	5	95	Ref	Ref		
**2. Hypertension**						
**Yes**	10	12	4.31[2.24-8.3]	13.4 [3.98-45.08]	2.6	<0.001
No	17	161	Ref	Ref		
**3. Sex**						
**Feminine**	22	98	3.37 [1.22-9.31]	5.84 [1.73-19.65]	1.76	**0.004**
Masculine	5	75	Ref	Ref		

F: Frequency. Ref: Reference. CI: Confidence interval. RR: Relative risk.

## Discussion

Mothers who conceived by IVF were aged, on average, 40.55 years old, and those who spontaneously conceived were 35.72 years old. Maternal age was significantly predominant in the exposed group compared to the unexposed group.

Indeed, this is explained by the fact that, from the age of 40 years old, the fertility rate of Cameroonian women tends to be at its lowest, almost close to zero, which is equal to 0.02.^10^ Consequently, this increases the risk of infertility. Among these women, pushing them more and more to access medically assisted procreation techniques such as IVF.

The results of Ezechi et al. 5 are similar to the present study's findings, reporting a similarly advanced age in mothers who have undergone IVF compared to those who conceived naturally. They reported a significant difference of 6 years between these two groups.

A significant predominance of maternal primiparity and abortion was reported in the exposed group compared to the unexposed group. In fact, the underlying explanation stems from the fact that from the age of 40, there is an accelerated decline in the production of quality ovum.^11^This leads to the increased formation of abnormal ova called aneuploid ova, which would be unable to be fertilised because of their chromosomal abnormalities. Even if fertilisation occurs, the resulting embryo carrying the abnormality would cause abortions.^12^ This is one of the reasons why the mothers of the exposed group, being older, were those who have had the most abortions in the past or the most likely to have never conceived before.

The results agree with those of Ezechi et al. in Nigeria,^5^ which identified a significant predominance of abortions in mothers of newborns from ART like IVF, compared with those of newborns from spontaneous conception (22% compared to 2.4%). On the contrary, the results of the study by Traoré et al. in Mali, 7 disagreed with those of the current study, revealing an equal and non-significant primiparity prevalence in the two groups of 49.78%. This could be because Traore et al., in their study, matched the two groups according to maternal parity.

This study's findings show a significant predominance of prematurity in newborns after IVF compared to those conceived spontaneously (22% and 5%, respectively). The exposed newborns were 4.4 times riskier to be born prematurely than those of the unexposed group. Other researchers have obtained results similar to that of this study.^6^,^7^,^13^-^18^ They all achieved a significantly higher prevalence of preterm birth after using ART like IVF, compared to spontaneously conceived births. Several theories could explain this predominance. First, the ageing of the woman would lead to ovarian functional disorders and vascular endothelial lesions, responsible for ovarian degeneration as well as a poor response to ovarian stimulators; 19,20subsequently, the type of transfer performed during the IVF procedure would also affect the prognosis for the weight of the newborn. Unlike cleavage and fresh transfers, blastocyst transfer and thaw-frozen transfer would lead to premature births.^21^

Moreover, in thawed frozen transfer cycles, the stimulated protocol, particularly the one based on estrogen-progesterone, would significantly increase neonatal prematurity (OR = 1.33, P<0.001) compared to the natural protocol.^21^ Furthermore, it is known that criteria such as the sex of the newborn, the maternal age at conception, and some diseases in pregnancy like hypertension, diabetes, and infectious diseases may expose the newborn to prematurity.^22^ But in this study, only the sex of the baby, arterial hypertension as a maternal disease in pregnancy, were significantly associated with prematurity.

And after controlling for these confounders, the risk of being born prematurely after IVF than after spontaneous conception was still significant.

These findings, however, are comparable to those of Gorgui et al.^13^, Dunietz et al.^14^, Marino et al.^17^, Hayashi et al.^18^ and Raatikainen et al.^23^ Indeed, they all found IVF as a significant risk factor of prematurity, while controlling the effects of the similar confounders as considered in the present study.

This study is similar to other studies, which assessed the risk of prematurity in infants conceived by IVF. 6,7,15,16It also shares similarities with other studies, which used nationally representative data in the analysis by extending the risk of occurrence of neonatal prematurity to other types of assisted reproduction techniques such as artificial insemination and intracytoplasmic sperm injection.^14^,^15^

However, there are also notable differences. This study differs from others in measuring gestational age, representing the term of pregnancy and defining premature birth. It used gestational age based on clinical estimation, as did Dunietz et al.^14^ However, some studies have used abdominal and vaginal ultrasound to assess the gestational age of newborns.^7^,^13^ Some studies, on the other hand, have used Dubowitz's criteria to estimate the gestational age after evaluating the neurological maturity of the premature baby.^6^

One of the strengths of this study is that, in line with a similar study by Barbuscia A et al.^15^, a retrospective cohort study was conducted. This meant prematurity was assessed in newborns conceived through in vitro fertilisation, representing the exposure factor. This was in contrast to similar studies, which instead employed case-control study designs with confusion about the notion of exposure (to IVF).^6^,^7^,^13^

Another strength is that this study excluded multiple pregnancies commonly known to result in premature births.^22^ Furthermore, after obtaining a significant prevalence of premature births in the exposed group, an additional analysis was performed by controlling the potential effect of other variables known to cause premature births, such as sex of the newborn, maternal age, some maternal disease in pregnancy like hypertension, diabetes, and infectious diseases. This was done to clearly highlight the effect of IVF on premature births. The low success rate of in vitro fertilisation in Cameroon, associated with the resulting limited access to newborns' medical records, explains that the sample size was not too large, counting as a weakness of this study.

This study showed that conducting in-vitro fertilisation is a risk factor for premature birth. It is, therefore, necessary to wonder if the damage caused by this technique, not yet well mastered in the context of this study, is only limited to prematurity or whether it could lead to other associated diseases. For example, it raises concerns about whether the methods of embryo transfer employed when performing in vitro fertilisation would not influence the abnormal aneuploid ova (common in infertile women older) and would not lead, in the event of pregnancy, to abnormal morphological and genetic modifications since egg would be resulting from an abnormal ovum. In other words, would IVF also lead to congenital and genetic diseases?

Thus, future research and researchers should consider in-depth and large-scale studies on the adverse effects of IVF on the health of newborns. It would also be important to investigate the different stages of this technique, which would be incriminated, to improve that technique and reduce or cancel those illnesses.

## Conclusion

This study reported a high predominance of prematurity in newborns from IVF compared to those born spontaneously. In addition, it clearly demonstrated that the risk of being born prematurely is higher in newborns conceived from IVF compared to those from spontaneous pregnancies, even after controlling for confounders. This shows that IVF is an absolute risk factor for premature births.
